# Polymorphonuclear Leukocyte Apoptosis Is Accelerated by Sulfatides or Sulfatides-Treated *Salmonella* Typhimurium Bacteria

**DOI:** 10.1155/2015/381232

**Published:** 2015-03-26

**Authors:** Zoryana V. Grishina, Galina M. Viryasova, Yulia M. Romanova, Galina F. Sud'ina

**Affiliations:** ^1^Belozersky Institute of Physico-Chemical Biology, Lomonosov Moscow State University, Moscow 119234, Russia; ^2^Gamaleya Research Institute of Epidemiology and Microbiology, Moscow 123098, Russia

## Abstract

Neutrophils die by apoptosis following activation and uptake of microbes or enter apoptosis spontaneously at the end of their lifespan if they do not encounter a pathogen. Here we report that sulfatides or sulfatides-treated *Salmonella* Typhimurium bacteria accelerated human neutrophil apoptosis. Neutrophil apoptosis was examined by flow cytometry. Sulfatides caused prominent increase in percentage of apoptotic cells after 2.5 hrs of incubation. *Salmonella* Typhimurium bacteria by themselves did not affect the basal level of apoptosis in neutrophil population. When neutrophils were added to *S.* Typhimurium “opsonized” by sulfatides, apoptotic index significantly increased, whereas the number of phagocyting cells was not influenced. Sulfatides' proapoptotic effect was strongly dependent on the activity of *β*-galactosidase; inhibition of this enzyme impaired its potency to accelerate apoptosis. These data support the mechanism of neutrophil apoptosis triggering based on sulfatides' ability to accumulate in intracellular compartments and mediate successive increase in ceramide content resulting from *β*-galactosidase activity.

## 1. Introduction

Neutrophils are programmed to undergo apoptosis, which limits their proinflammatory potential. Neutrophil's prolonged survival contributes to tissue damage. Apoptosis would prevent release of cytotoxic products such as reactive oxygen species (ROS) or proteases that contribute to tissue damage.

Neutrophils play a key role during the early phases of infection. Human polymorphonuclear leukocytes (PMNLs, neutrophils) can kill bacteria. In 1996 Watson et al. discovered that phagocytosis of* E. coli* induced neutrophil apoptosis [1]. Following phagocytosis of bacteria, neutrophils produce ROS and degranulate. These processes destroy ingested microorganisms, after which neutrophils undergo apoptosis and are removed by macrophages, which help resolve infection.

Several bacterial factors, including lipopolysaccharides and lipoteichoic acid, inhibit neutrophil apoptosis [2]. Neutrophil apoptosis following phagocytosis of pathogens is beneficial to the host, so we looked for the factors that can affect it.

Sulfatides are one of the major sphingoglycolipids in mammalian serum and are synthesized and secreted mainly from the liver as a component of lipoproteins [3]. Serum sulfatides count is 10 *μ*M and higher in human blood [4] and 1.5 *μ*M in mouse blood [5]. Recent studies revealed a protective role for serum sulfatides against arteriosclerosis and hypercoagulation [6]. In the cerebrospinal fluid, lipoprotein particles contain large amount of sulfatides and modulate sulfatide homeostasis in the central nervous system via endocytotic pathways [7]. Accumulation of sulfatides in the cell can induce neuronal cell apoptosis [8].

Sulfatides play a role in bacteria's interaction with epithelial cells. Epithelial cells are responsible for the release of a variety of factors that attract neutrophils and coordinate the immune response to microbes. To infect a host, bacteria need contact with the epithelial surface (respiratory or intestine). Sulfatides act as binding molecules for the attachment of* M. catarrhalis* to human pharyngeal epithelial cells, and synthetic sulfatides inhibit the attachment of* M. catarrhalis* significantly at an optimum concentration of 10 *μ*g/mL [9]. Sulfatides mediate the attachment of* Pseudomonas aeruginosa* to human epithelial cells [10]. Immunohistochemistry study detected sulfatides on the surface of mucosal epithelial cells, involved in the binding of* Escherichia coli* enterotoxin b [11, 12].

When neutrophils migrate across the epithelial paracellular junction into the lumen, they can interact with sulfatides on the brush border epithelial cell surface as well as encounter bacteria, which might have sulfatides on their surface.

It has been known that infection with* S.* Typhimurium is associated with the rapid loss of barrier function of epithelial monolayer and rapid PMNL migration across the intestinal barrier to the epithelial surface where salmonella bacteria directly invade intestinal epithelial cells [13, 14]. To clarify the ability of sulfatides to influence PMNL-bacteria interaction, we studied* S.* Typhimurium cells “opsonized” by sulfatides. The aim of our study was to evaluate the role of sulfatides in neutrophil survival and in neutrophil interaction with bacteria* Salmonella* Typhimurium. Our results showed that sulfatides accelerated apoptotic death of neutrophils.

## 2. Materials and Methods

*S.* Typhimurium virulent strain C53 was a kind gift from Professor Norel (Pasteur Institute, France) [15]. We grew bacteria in Luria-Bertani broth and washed them twice in physiological salt solution with centrifugation at 2000 g. The concentration of the stock suspension was 1 × 10^9^ CFU/mL. Sulfatides and HEPES were from Fluka (Deisenhofen, Germany). We dissolved sulfatides at 50 mg/mL in a mixture of ethanol/DMSO (1 : 3). Carboxyfluorescein Multicaspase Activity Kit (AK-117) was from BIOMOL (Plymouth Meeting, PA). Annexin V-Alexa Fluor 488 Conjugate, Annexin V-Alexa Fluor 647, and propidium iodide (PI) were from Molecular Probes (Mo Bi Tec, Göttingen, Germany). Cell-permeant  *β*-galactosidase inhibitor Phenylethyl  *β*-D-thiogalactopyranoside (PETG) was from Invitrogen (Carlsbad, CA). Ficoll-Paque was from Pharmacia (Uppsala, Sweden). Phosphate buffered saline (PBS) was from Gibco (Paisley, UK; Scotland, UK). Dextran T-500 was from Pharmacosmos (Holbaek, Denmark). Hank's balanced salt solution (with calcium and magnesium but without phenol red and sodium hydrogen carbonate, HBSS), HBSS modified (without calcium, magnesium, phenol red, and sodium hydrogen carbonate), Dulbecco's PBS (with magnesium but without calcium), cytochalasin D, bovine serum albumin, fraction V (BSA), and propidium iodide (PI) were from Sigma-Aldrich Chemie GmbH (Steinheim, Germany).

### 2.1. Human Neutrophil Isolation

For neutrophil preparation we used the blood of healthy volunteers. Blood was taken via venous puncture as approved by Ministry of Public Health Service of Russian Federation. The Institutional Ethics Committee of the A. N. Belozersky Institute of Physico-Chemical Biology, Moscow State University, approved blood experimental procedures and the consent procedure. We isolated PMNLs from freshly drawn citrate-anticoagulated donor blood by standard techniques, as previously described [16]. We prepared leukocyte-rich plasma by sedimentation of red blood cells with 3% dextran T-500 at room temperature. We purified granulocytes by centrifugation of leukocyte-rich plasma through Ficoll-Paque (density 1.077 g/mL) followed by hypotonic lysis of the remaining RBCs. We washed PMNLs twice with PBS, resuspended at 10^7^/mL (purity 96-97%, viability 98-99%) in Dulbecco's PBS containing 1 mg/mL glucose (without CaCl_2_), and stored at room temperature.

### 2.2. Preparation of Opsonized* S.* Typhimurium: Pretreatment of* S.* Typhimurium with Sulfatides

The bacteria were washed twice to remove growth medium in PBS, opsonized with 5% normal serum (NS) and/or treated with 250 *μ*g/mL sulfatides for 15 min at 37°C, centrifuged, and resuspended in Hank's balanced salts medium containing 10 mM Hepes (HBSS/Hepes).

### 2.3. Detection of PMNLs Phagocytosis of* S.* Typhimurium by Flow Cytometry

We followed the method reported by Hatano et al. [17] with some modifications. Briefly, we heat-killed bacteria by incubation at 60°C for 40 min, washed them twice, and resuspended them in PBS containing 5 mg/mL propidium iodide. We incubated the suspension in the dark at room temperature for 2 h. The fluorescent bacteria were washed three times in PBS and opsonized with 5% NS or treated with 250 *μ*g/mL sulfatides for 15 min at 37°C. We then washed them in HBSS/Hepes and added them to neutrophil suspension at a ratio of 10 bacteria/1 neutrophil (finally diluted as 1 : 10). Samples were incubated in the dark for 15 min at 37°C. After incubation, centrifugation pelleted the neutrophils and we washed them twice in PBS to remove nonphagocytized bacteria. We analyzed samples immediately with a Cytomics FC 500 Flow Cytometry System (Beckman Coulter, Germany) with CXP software and collected red fluorescence through a 620 nm long-pass filter. For each acquisition, at least 10,000 events were collected within 90 s of measurement.

### 2.4. Apoptosis Detection by Flow Cytometry

We suspended PMNLs at a density of 1.5 × 10^6^ cells per mL in HBSS/Hepes and added or not with tested compounds (sulfatides, 10 *μ*M cytochalasin D, 3 mM PETG; cytochalasin D and PETG were added 30 min prior sulfatides), or with opsonized with normal serum or sulfatide-treated* S.* Typhimurium (at a ratio of 10 bacteria/1 neutrophil, finally diluted as 1 : 10). Cells incubated for 2.5 h at 37°C before the addition of Annexin V-Alexa Fluor commercial solution at a 1/100 dilution. After 10 min at room temperature, centrifugation pelleted the neutrophils and we washed them in HBSS/Hepes and then we analyzed samples immediately using a Cytomics FC 500 Flow Cytometry System (Beckman Coulter, Germany) with CXP software and red fluorescence was collected through a 680 nm long-pass filter. For each acquisition, at least 10,000 events were collected within 90 s of measurement. In our preliminary experiments we found that after 2.5 h incubations both* S.* Typhimurium bacteria and zymosan particles give high background fluorescence, which strongly interfered with fluorescence of Annexin V-Alexa Fluor 488 Conjugate, and just low background with Annexin V-Alexa Fluor 647, so we used the latter to stain phosphatidylserine expression on apoptotic cells.

### 2.5. Caspases Activation Detection by Flow Cytometry

Caspases activation was detected using commercial BIOMOL Carboxyfluorescein Multicaspase Activity Kit in accordance with the manufacturer's recommendations. Briefly PMNLs were suspended at a density of 1.5 × 10^6^ cells per mL in HBSS/Hepes and we added 25 *μ*g/mL sulfatides or did not. Cells incubated for 1.5 h at 37°C. We added aliquots of staining solution to the samples for 1 h. Then centrifugation pelleted the neutrophils and we washed them in HBSS/Hepes. We analyzed samples immediately with a Cytomics FC 500 Flow Cytometry System (Beckman Coulter, Germany) with CXP software and collected green fluorescence. Each acquisition collected at least 10,000 events within 90 s measurement.

### 2.6. Statistics

Statistical analysis was performed using Student's *t*-test. Statistical significance was assumed, where probability of less than 0.05 was obtained. Results are reported as mean ± SD of the data of at least three independent experiments. Statistically significant differences are indicated with one asterisk (*P* < 0.05) or two (*P* < 0.01).

## 3. Results

### 3.1. Acceleration of Neutrophil Apoptosis by Sulfatides

**Figure 1:**
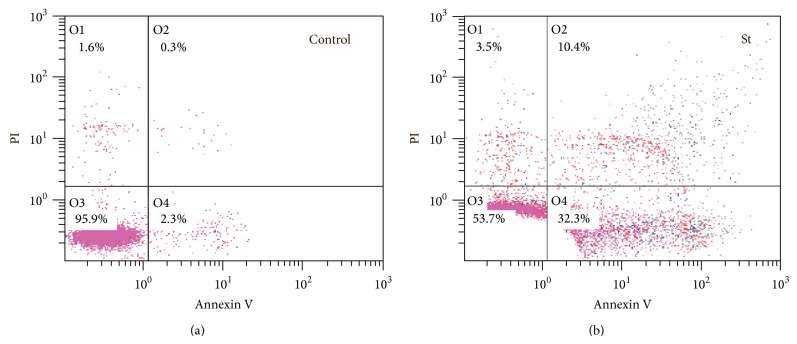
Dot plots for phosphatidylserine externalization of the apoptotic cells. We treated PMNLs (1.5 × 10^6^/mL) for 2.5 h at 37°C without (a) or with (b) 25 *μ*g/mL sulfatide (St) and stained them with Annexin V-Alexa Fluor 488 and PI and processed them by flow cytometer as described in Section 2. The percentage of viable cells (region O3), early apoptotic cells (region O4), and late apoptotic and necrotic cells (regions O2 and O1) is indicated. Representative dot plots are shown from four independent experiments.

PMNLs incubated with no addition or in the presence of sulfatides, and then we analyzed exposure of phosphatidylserine (PS) by detection of membrane-bound Annexin V-Alexa Fluor fluorescence, and plasma membrane integrity loss was analyzed using propidium iodide (PI). At 2.5 h incubation most of the cells (>96%) retained an intact membrane, as determined by Alexa Fluor 488 and PI staining (Figure 1). We consider the data for Annexin V staining in (Annexin V^+^/PI^−^) cells to be representative, and further we present the data for Annexin V-Alexa Fluor 647 fluorescence.

**Figure 2:**
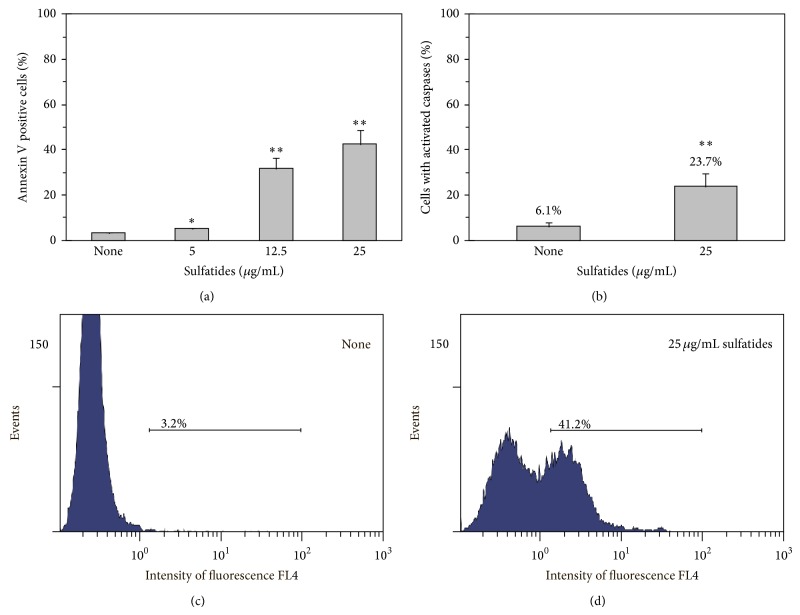
Evaluation of sulfatide-induced PMNL apoptosis. (a), (c), and (d) PS exposure of PMNL was determined using Annexin V-Alexa Fluor 647. PMNLs (1.5 × 10^6^/mL) were cultured for 2.5 h at 37°C without or with sulfatides (indicated concentration). Then, flow cytometry determined the percentage of Annexin V positive cells. Shown are the mean ± SD (a) and flow cytometric histogram samples of baseline (c) and 25 *μ*g/mL sulfatide (d) groups, from six independent experiments. (b) PMNLs (1.5 × 10^6^ cells/mL) were added or not added with 25 *μ*g/mL sulfatides, and, after 1.5 h at 37°C, the BIOMOL Carboxyfluorescein Multicaspase Activity Kit used flow cytometry to count the cells with activated caspases. Shown is the mean ± SD of three independent experiments.

Sulfatides induced PMNLs apoptosis, as evidenced by PS externalization (Annexin V positive cells, Figures 1 and 2) and caspase activation (Figure 2(b)). We observed well-distinguishable increase in the percentage of apoptotic cells after 2.5 hours of treatment with 5 *μ*g/mL sulfatide. Sulfatides' proapoptotic effectiveness was dose-dependent. At 25 *μ*g/mL sulfatides the number of Annexin V positive cells in the population exceeded 40%.

Using Carboxyfluorescein Multicaspase Activity Kit (BIOMOL), we revealed increasing caspase activation in neutrophils treated with sulfatides. Our data indicate that the number of the cells with active caspases increased significantly (*P* < 0.01) with 25 *μ*g/mL sulfatides.

### 3.2. Sulfatides Accelerated Apoptosis of PMNLs Incubated with* Salmonella enterica* serovar Typhimurium (*S.* Typhimurium) Bacteria

We further used* Salmonella enterica* serovar Typhimurium (*S.* Typhimurium) bacteria to determine whether sulfatides could influence apoptosis of neutrophils following phagocytic interaction with bacteria. To test the role of sulfatides in PMNL-bacteria interaction, we “opsonized” bacteria by sulfatides.

**Figure 3:**
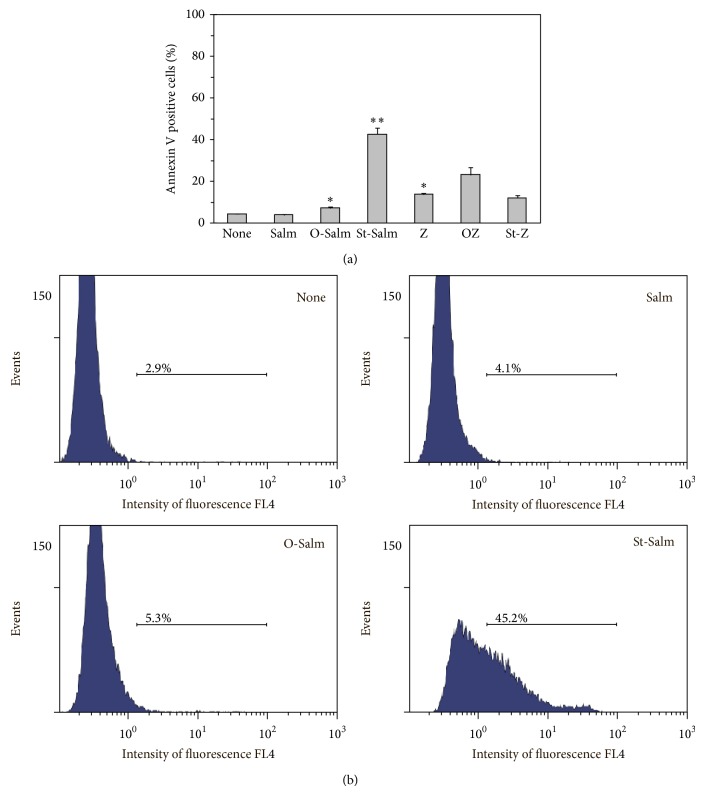
Assay of PMNL apoptosis developed in probes added with* S.* Typhimurium bacteria, after 2.5 hours of coincubation. Neutrophils were challenged with plain bacteria (Salm), opsonized with normal serum bacteria (O-Salm), and treated with sulfatides bacteria (St-Salm), as described in Section 2. We also present data of similar experiments in which zymosan—nonopsonized (Z), opsonized by normal serum (OZ) or treated with sulfatides (St-Z)—provided phagocytosable stimulus of PMNLs. Then, PS exposure of PMNL was determined using Annexin V. Shown are the mean ± SD (a) and representative spectra (b) of five independent experiments.

The number of apoptotic cells in probes with nonopsonized bacteria (Salm) was similar to that of control probes with untreated neutrophils (Figure 3). Opsonized by normal serum bacteria (O-Salm) caused twofold increase of apoptotic cells above Salm probes and basal level of apoptosis giving the value of less than 10% (Figure 3(a)). The number of Annexin V positive neutrophils in probes, added with* S.* Typhimurium “opsonized” by sulfatides (St-Salm), was crucially raised up exceeding 40%.

Similar experiments we performed with zymosan are as follows: nonopsonized (Z), opsonized by normal serum (OZ), or treated with sulfatides (St-Z). PMNL activation with untreated or treated zymosan resulted in the remarkable apoptotic response: up to 15% with Z and St-Z and up to 25% with OZ (Figure 3(a)). It is noteworthy that we did not observe any difference between Z and St-Z probes. Thus sulfatides are not able to influence apoptosis of neutrophils phagocyting zymosan which is not really a physiologically relevant phagocytosable stimulus like bacteria are.

**Figure 4:**
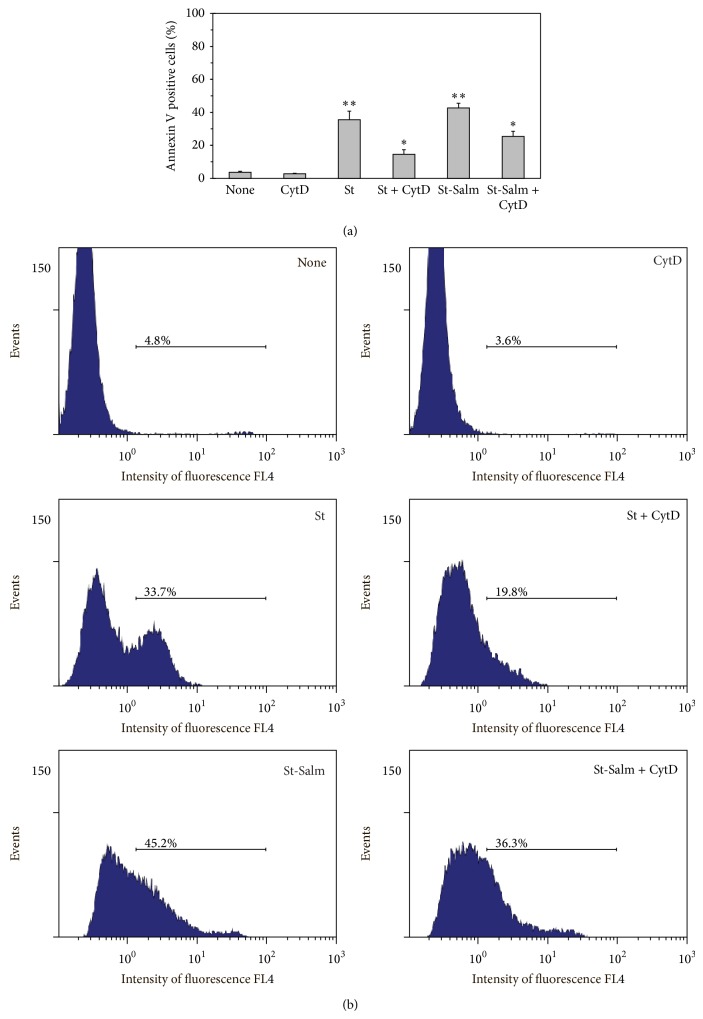
Effect of Cytochalasin D on PMNL apoptosis. We cultured PMNLs (1.5 × 10^6^/mL) for 2.5 h at 37°C without or with 25 *μ*g/mL sulfatides (St) or challenged with sulfatides-treated bacteria (St-Salm), and/or 10 *μ*M Cyto D, as indicated. Shown are the mean ± SD (a) and representative flow cytometric spectra (b) of four independent experiments.

Blocking* S.* Typhimurium phagocytosis with cytochalasin D, we observed a distinguishable decline of sulfatides' proapoptotic effect. However, similar partial abrogation of sulfatide influence by actin polymerization inhibitor was detected in probes where PMNLs were challenged with sulfatides without bacteria, that is, when apoptosis did not result from phagocytosis (Figure 4). The inhibitor of actin polymerization and endocytosis cytochalasin D caused more than a 50% reduction of sulfatide proapoptotic effects.

### 3.3. Phagocytosis of* S.* Typhimurium by PMNLs

**Figure 5:**
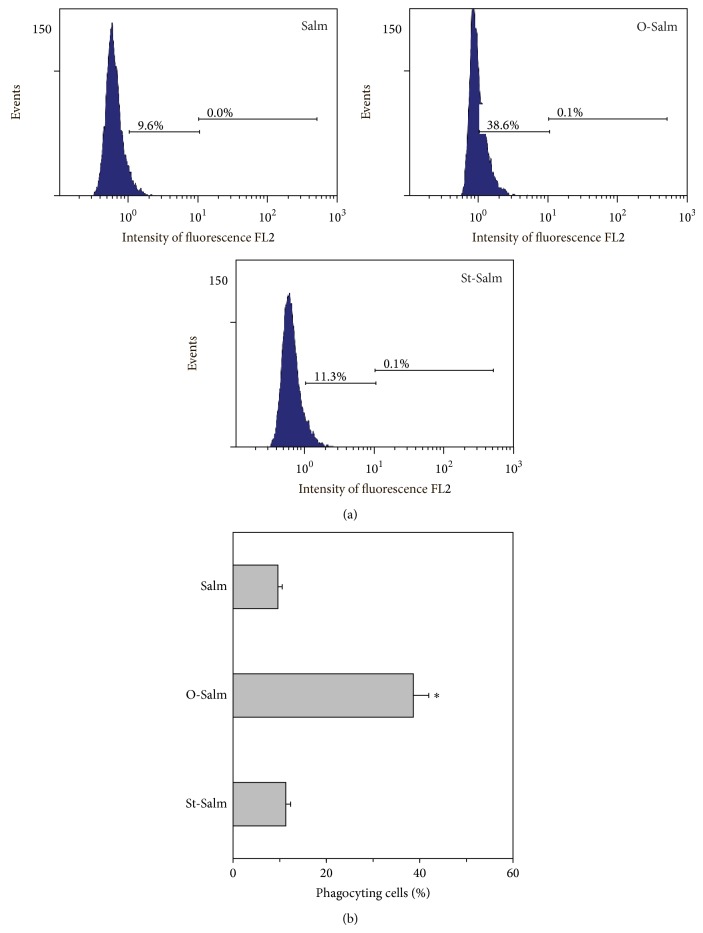
Phagocytosis of* S.* Typhimurium by PMNLs. We added PMNLs with plain bacteria (Salm), opsonized by normal serum (O-Salm) or treated by sulfatides (St-Salm) at a ratio of 10 bacteria/1 neutrophil. We assessed percentage of phagocyting cells by flow cytometry after 15 min of incubation of PMNLs with bacteria and removal of nonphagocytized bacteria. Shown are the mean ± SD (b) and representative flow cytometric spectra (a) of three independent experiments.

We performed several series of experiments to analyze phagocytosis of* S.* Typhimurium by PMNLs. The level of phagocytic activity of the cells in both Salm and St-Salm probes was comparable and could be characterized as low (Figure 5). As expected, ingestion of O-Salm by neutrophils was much more active: the percentage of phagocyting cells was determined as near 40%. Thus, we could not see any correlation of apoptotic cell number in probes added with St-Salm and number of the cells phagocyting St-Salm.

This fact in addition to our data demonstrating that sulfatide-induced acceleration of apoptosis is suppressed by Cyto D may point out that sulfatide proapoptotic effectiveness is mediated by sulfatide delivery to the cell. We reported previously that sulfatides are able to rapidly penetrate into cells, and the lipid incorporation was abolished by cytochalasin D [18]. Our results demonstrate that this sulfated lipid directs PMNLs to undergo apoptosis when the cells are exposed to sulfatides or sulfatides-treated* Salmonella* bacteria.

### 3.4. Inhibition of *β*-Galactosidase Attenuates Sulfatide-Induced Apoptosis

**Figure 6:**
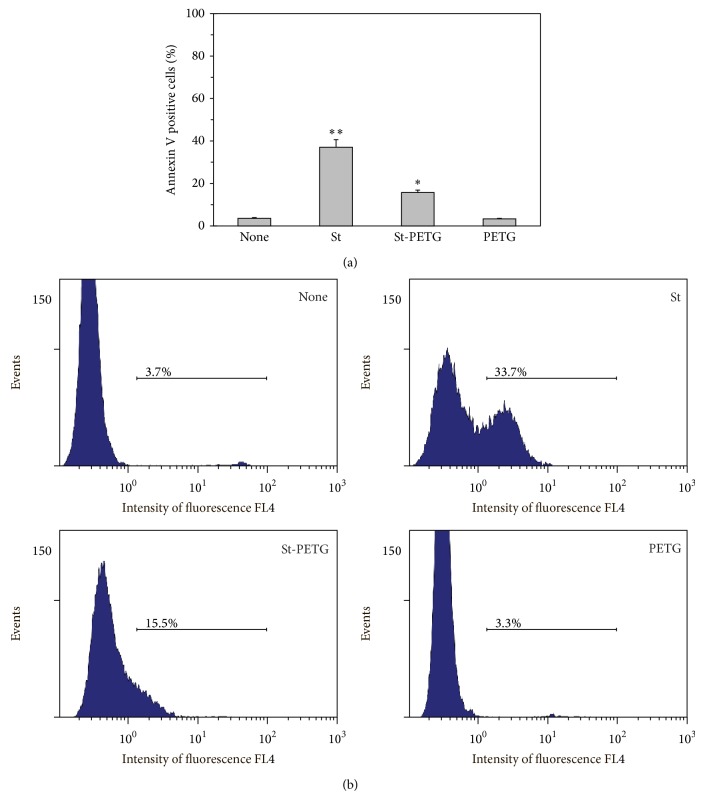
*β*-galactosidase inhibitor PETG attenuates sulfatide-induced apoptosis. We cultured PMNLs for 2.5 h at 37°C without or with tested compounds (25 *μ*g/mL sulfatides, 3 mM PETG; PETG was added 30 min prior to sulfatides). Then flow cytometry determined the percentage of Annexin V positive cells. Shown are the mean ± SD (a) and representative flow cytometric spectra (b) of four independent experiments.

The hydrolysis of sulfatide to ceramide and accumulation of* ceramide* species in the cells were revealed as a pivotal biochemical mechanism of sulfatide-induced apoptosis of neuronal cells [8]. *β*-galactosidase activity is responsible for direct hydrolysis of sulfatide to ceramide without prior desulfation. In the experiments using the cell-permeable *β*-galactosidase inhibitor PETG (based on Dr. P. Held, BioTek Instruments, Inc., http://www.biotek.com/resources/articles/kinetic-analysis-beta-galactosidase.html), we observed prominent decline of sulfatide-induced apoptosis of PMNLs (Figure 6). These data support sulfatide action in neutrophils which involves* ceramide* accumulation provided by *β*-galactosidase conversion of sulfatide. To test whether another substrate of *β*-galactosidase could promote apoptosis, we used nonsulfated galactocerebroside (GalCer) instead of sulfatide; however, we did not observe any effect of GalCer on PMNL apoptosis (data not shown).

## 4. Discussion

Apoptosis is a very essential mechanism in the regulation of neutrophil homeostasis and inflammation. Apoptotic death of neutrophils helps resolution in many ways. Injection of apoptotic neutrophils protected mice against lipopolysaccharide-induced shock [19]. Apoptotic cells can also protect one from endotoxic shock via direct binding and removal of LPS [19]. Accelerated or delayed neutrophil apoptosis can have severe pathological consequences including cystic fibrosis [20], asthma [21], and sepsis [22, 23]. Accelerating apoptosis, intracellular pathogens may use apoptotic neutrophils as a Trojan horse to infect other cells [24–26]. As discovered recently, neutrophils engulf* S.* Typhimurium right in the gut lumen and keep them there for a limited time [27]. Sulfatide is one of the glycolipid receptors that stick bacteria to the mucosal surface.* M. pneumonia* [28] and* P. aeruginosa* use sulfatide to adhere directly to the host respiratory tract [10]. As sulfatides play a role in bacterial interaction with epithelial cells, we aimed in this study to characterize PMNL-bacteria interaction as influenced by sulfatides. Given the complexity of pathways involved in the regulation of neutrophil apoptosis, we only looked on the net apoptotic response to sulfatides or sulfatide-treated* Salmonella*.

Sulfatides (3-O-sulfogalactosylceramides, sulfated galactocerebrosides, or SM4) are sphingolipids found at the extracellular leaflet of the plasma membrane of most eukaryotic cells. They are expressed predominantly in the myelin sheath of the nervous system [3] but also found at the surface of blood cells [29–32] and as a component of lipoproteins in blood serum [33]. Toxins from pathogenic organisms recognize sulfatide at the cell surface to mediate toxin endocytosis and enhance the virulence of the pathogen [34–37]. In its turn, sulfatide administration (adding of exogenous sulfatide) prevents cell infection with viruses [38–41]. Efficient propagation of influenza A virus (IAV) was observed in cells with genetically increased sulfatide synthesis [42]. Sulfatide-enriched COS7 cells were highly susceptible to apoptosis and showed an increase in IAV replication. The role of sulfatides is clearly emerging and many questions about mechanisms of their action and signaling need to be addressed.

Our results demonstrate that apoptotic cell death is accelerated in human neutrophils after their exposure to sulfatides or sulfatide-treated* Salmonella* bacteria. Sulfatides promoted activation of caspases in neutrophils. Their proapoptotic effects depended on the activity of *β-*galactosidase, and inhibition of this enzyme abolished sulfatide-induced apoptosis of neutrophils. When the sulfolipid was introduced to the cells being incorporated into the object of phagocytosis (St-Salm), apoptosis was drastically augmented. It is noteworthy that sulfatides had no effect on apoptosis of PMNLs exposed to zymosan. As zymosan is a potent activator of PMNL apoptosis by itself, the impact of sulfatides was probably indistinguishable on this background.

Our current study presents results on the abolition of the sulfatide effects on neutrophil apoptosis. We used two kinds of effectors for this abolition: cytochalasin D, which prevents sulfatide internalization into the cells [18], and cell-permeable *β*-galactosidase inhibitor PETG, a competitive inhibitor of sulfatide transformation to ceramide. Our results confirmed that the proapoptotic effectiveness of sulfatides depends on their ability to accumulate in intracellular compartments and mediate successive increase in ceramide content resulting from  *β*-galactosidase activity.

The physiological significance of sulfatides in regulation of neutrophil function is not clearly revealed. Our data suggest that sulfatides by themselves or incorporated into bacterial membranes crucially accelerate neutrophil apoptosis. Such mechanism may help explain the diminished accumulation of neutrophils in inflamed tissues observed in studies* in vivo* with these substances [43, 44].
